# Aesthetic Surgery Fellowship at Akademikliniken Stockholm: Survey among Graduates

**DOI:** 10.1007/s00266-024-03939-w

**Published:** 2024-03-27

**Authors:** Paolo Montemurro, Yannick F. Diehm, Per Hedén, Sebastian Fischer, Johannes M. Wagner

**Affiliations:** 1 Akademikliniken Stockholm, Storängsvägen 10, 11541 Stockholm, Sweden; 2 Department of Surgery, Division of Plastic Surgery, Brigham and Women’s Hospital, Harvard Medical School, 75 Francis St, Boston, 02115 USA; 3 Department of Plastic and Reconstructive Surgery, BG Clinic Ludwigshafen, University of Heidelberg, Ludwig-Guttmann-Straße 13, 67071 Ludwigshafen, Germany; 4 Department of Plastic, Reconstructive and Hand Surgery, BG University Hospital Bergmannsheil Bochum, Bürkle-de-la-Camp Platz 1, 44789 Bochum, Germany

**Keywords:** Fellowship, Aesthetic surgery, Education, Breast surgery, Breast augmentation

## Abstract

**Background:**

Aesthetic Surgery is one of the most competitive fields of plastic surgery. Although there is a certain demand for highly educated surgeons in this field, training in cosmetic procedures remains challenging. Akademikliniken Stockholm offers a highly appreciated fellowship program for aesthetic plastic surgeons and trained more than 200 surgeons from all over the world.

**Objectives:**

The aim of the present work was to provide insights into this fellowship program, analyze what graduates have learned and if this had implications on their further professional orientation.

**Methods:**

Participants of the Akademikliniken fellowship program, who graduated between 10/2008 and 10/2018 (*n *= 66) were invited to take part in an online survey which included 30 questions about general demographics and about experience before, during and after the fellowship.

**Results:**

Thirty-four graduates participated in the survey (52%). Twenty-four graduates (71%) had been already specialists in plastic surgery before commencing the fellowship program. Mean length of fellowship was 7 months (range 3–24months). Numbers of aesthetic procedures performed by the applicants significantly increased after the fellowship, and moreover, the scope of daily clinical practice shifted toward aesthetics in almost all applicants.

**Conclusions:**

A well-designed dedicated aesthetic surgery fellowship can improve the lack of training, aesthetic surgeons have during their residency. Graduates of our fellowship program reported great improvements in confidence in performing aesthetic procedures and a benefit for their future career.

**Level of Evidence III:**

This journal requires that authors assign a level of evidence to each article. For a full description of these Evidence-Based Medicine ratings, please refer to the Table of Contents or the online Instructions to Authors www.springer.com/00266.

## Introduction

Aesthetic surgery is a challenging yet rewarding discipline and probably the most competitive field in plastic and reconstructive surgery. According to the International Society of Aesthetic Plastic Surgery, the number of aesthetic procedures rises continuously, reaching an all-time high in 2018 with more than 10.5 million surgeries worldwide [1]. Although this clearly indicates an increasing demand for doctors who are educated in cosmetic surgery, receiving training in such procedures remains challenging [2–6]. Aesthetic surgeries are usually a bottleneck during residency. Most departments for plastic and reconstructive surgery that are eligible to train residents focus on reconstruction rather than aesthetics and thus show relatively low numbers of aesthetic procedures available for teaching purposes [4]. In contrast, private practices have almost only cosmetic patients but they are usually too small to host a trainee and too dependent on economic efficiency to spend time with teaching. Therefore, fellowships in clinics with higher capacities and focus on aesthetic surgery are a recommended way to learn and gain experience in cosmetic procedures [7–9].

Established in 2005 and currently organized by the corresponding author (paolo.montemurro@ak.se), the fellowship program at Akademiklinken Stockholm has trained more than 200 surgeons from all over the world. With the tremendous efforts in teaching and by spreading the spirit of the motto *beauty through science*, we believe that the fellowship program at our institution has provided high-quality education and has a significant impact on the future career of the graduates. As a matter of fact, many of these graduates have been head hunted by private clinics after completing their fellowship and are now working as cosmetic surgeons.

The aim of this study was to provide insights into our fellowship program, analyze what graduates have learned and if this had implications on their further professional orientation.

## Materials and Methods

Participants of the Akademikliniken fellowship program, who graduated between 10/2008 and 10/2018 (*n *= 66) were invited to take part in an online survey (www.surveymonkey.com). Invitations were sent by email. Emails included a link to the survey that did not retrieve any identifying information to facilitate anonymous participation. To further promote study participation, a reminder email was sent 2 weeks later.

**Fig. 1:**
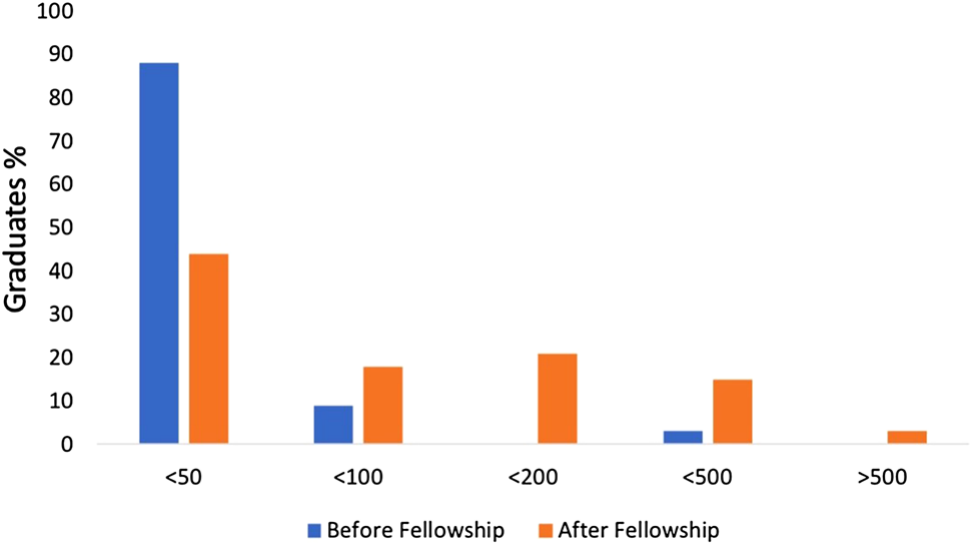
Numbers of aesthetic surgeries performed by the graduates before and after completion of the fellowship program. Results are given in percent. Asterix denote statistical significance between before and after fellowship responses

The survey included 30 questions separated into general demographic questions, and questions about experience before, during and after the fellowship (Table 1). Type of questions involved closed-ended (Q4, Q12–13, Q28–30), open-ended (Q5, Q7), as well as single (Q6, Q10–11, Q14–22, Q24–27) and multi select multiple choice questions (Q8–9, Q23). General demographic questions addressed age (Q1), gender (Q2), nationality (Q3), specialist status before fellowship (Q4), duration of fellowship (Q5), year of graduation from fellowship (Q7), current specialist status (Q10), current position (Q11), and current institution (Q12) (Tables 2 and 3, Fig. 1).

**Table 1:** Survey questionnaire

*Demographic questions*			
Q1	Gender	Male/Female	
Q2	Age	Open	
Q3	Nationality	Open	
Q4	Before fellowship, have you already been a specialist in plastic surgery?	Yes/no	
Q5	For how long have you been fellow at AK?	Open	
Q6...	The duration of your fellowship was	Too short/exactly right/too long	
Q7	In which year did your fellowship end?	Open	
Q10	What is your current postgraduate year (PGY) or for how many years are your currently board certified?	PGY2/3/4/5/6/> 6/board certified < 1 y/< 2 y/< 3 y/> 3 y	
Q11	What is your current position?	Resident/attending/chief/private practice/mixed practice	
Q12	Are you currently employed at the same institution/private practice than before the fellowship?	Yes/no	
Q13	If no, how many years after fellowship completion did you change your institution?	< 1 y/< 2 y/> 2 y	
*Questions about experience before fellowship*			
Q8	What was the scope of your daily practice before fellowship?	Aesthetics/burns/reconstructive/hand/others (multiple answers possible)	
Q18	How many aesthetic surgeries per year did you perform before the fellowship?	<= 50/51–100/101–200/201–500/> 500	
Q19	Your experience with aesthetic surgeries before the fellowship was..	Too low/exactly right/too high	
Q21	Please rate your level of confidence with the following aesthetic surgery procedures before fellowship?	Facelift/blepharoplasty/rhinoplasty/breast augmentation/mastopexy/augmentation mastopexy/breast reduction/abdominoplasty/brachioplasty/liposuction (scale 1–5, 1 = not confident, 5 very confident)	
Q26	How many papers did you publish before the fellowship?	< 5/< 10/< 30/< 50/> 50	
*Questions about the fellowship*			
Q24	What was your motivation to apply for the fellowship?	Aesthetics/research/both/other	
Q9	How did you finance your fellowship?	By home clinic/practice/by AK/savings/debt (multiple answers possible)	
Q15	In how many of the following procedures did you participate during your fellowship?	Blepharoplasty	0/1–5/6–20/21–50/> 50
		Facelift	0/1–5/6–20/21–50/> 50
		Rhinoplasty	0/1–5/6–20/21–50/> 50
		Breast augmentation	0/1–5/6–20/21–50/> 50
		Augmentation mastopexy	0/1–5/6–20/21–50/> 50
		Mastopexy	0/1–5/6–20/21–50/> 50
		Capsulectomy	0/1–5/6–20/21–50/> 50
Q14	What was the most interesting field at Akademikliniken?	Breast/face w/o nose/nose/body contouring/botox/filler	
*Questions about experience after fellowship*			
Q16	How many of the surgical techniques introduced to you at Akademikliniken could you implement into your daily practice?	< 10%/< 25%< 50%/< 75%/<= 100%	
Q17	Did the amount of aesthetic surgeries in your daily practice alter in the first two years after the fellowship?	Decreased/same/increased < 25%/increased < 50%/doubled/> doubled	
Q20	How many aesthetic surgeries per year do you currently perform?	< 50/< 100/< 200/< 500/> 500	
Q22	Please rate your level of confidence with the following aesthetic surgery procedures after fellowship	Facelift/blepharoplasty/rhinoplasty/breast augmentation/mastopexy/augmentation mastopexy/breast reduction/abdominoplasty/brachioplasty/liposuction (scale 1–5, 1 = not confident, 5 very confident)	
Q23	What is the scope of your daily practice today?	Aesthetics/hand/burns/reconstruction/other	
Q25	Did your interest in research alter after the fellowship?	Decreased/same/increased	
Q27	How many papers did you publish to date?	< 5/< 10/< 30/< 50/> 50	
Q28	Did the fellowship fulfill your expectations?	Yes/no	
Q29	Did the fellowship push your career?	Yes/no	
Q30	Would you recommend the fellowship?	Yes/no

**Table 2:** Participant demographics

Participants and response rate	*n *= 34 (54%)
Gender	
Male	*n *= 30 (88%)
Female	*n *= 4 (12%)
Country of origin	
Australia	*n *= 1 (3%)
Austria	*n *= 1 (3%)
Cyprus	*n *= 1 (3%)
Iceland	*n *= 1 (3%)
Litany	*n *= 1 (3%)
Montenegro	*n *= 1 (3%)
Romania	*n *= 1 (3%)
San Marino	*n *= 1 (3%)
Turkey	*n *= 1 (3%)
India	*n *= 1 (3%)
Brazil	*n *= 2 (6%)
Sweden	*n *= 2 (6%)
Germany	*n *= 4 (12%)
Greece	*n *= 5 (14%)
United Kingdom	*n *= 5(14%)
Italy	*n *= 6 (18%)
Plastic surgery specialists	*n *= 24 (71%)
Mean duration of fellowship	7 (3–24) months
Mean time from end of fellowship to survey	43 (3–120) months

Results of demographic survey questions including gender, country of origin, mean fellowship duration, time between end of fellowship and survey participation as well as board-specification

**Table 3:** Number of surgeries during fellowship

	0	1–5	6–20	21–50	> 50
Blepharoplasty	0 (0)	0 (0)	16 (47.06)	13 (38.24)	5 (14.71)
Facelift	0 (0)	4 (11.76)	18 (52.94)	11 (32.35)	1 (2.94)
Rhinoplasty	0 (0)	1 (2.94)	17 (50)	10 (29.41)	6 (17.65)
Breast augmentation	0 (0)	0 (0)	1 (2.94)	13 (38.24)	20 (58.82)
Augmentation mastopexy	0 (0)	2 (5.88)	12 (35.29)	12 (35.29)	23.53 (8)
Mastopexy	0 (0)	3 (8.82)	12 (35.29)	15 (44.12)	4 (11.76)
Capsulectomy	0 (0)	8 (23.53)	17 (50)	9 (26.47	0 (0)

Results of Q15. Number of graduates who participated in 0, 1–5, 6–20, 21–50 and > 50 of key aesthetic procedures during their fellowship. Results are given as absolute numbers n and percent (%).

Questions addressing experience before the fellowship focused on specialist status (Q4, yes/no), scope of daily practice (Q8, aesthetic/hand/burns/reconstruction/others), number of (Q18, <= 50/51–100/101–200/201–500/> 500) and confidence with aesthetic surgeries (Q21; scale 1 to 5; 1 = not confident, 5 = very confident). The latter was asked specifically for the procedures facelift, blepharoplasty, rhinoplasty, breast augmentation, mastopexy, augmentation mastopexy, breast reduction, abdominoplasty, brachioplasty and liposuction. Last, participants were asked for the number of scientific papers they had published annually before the fellowship (Q26; <= 5/6–10/11–30/31–50/> 50). Questions about the fellowship involved financing (Q9; by home clinic/practice/by Akademikliniken/by savings/by debt), motivation for application (Q24; aesthetics/research/both/other), as well as number and type of procedures participated in (Q15; 0/1–5/6–20/21–50/> 50). The latter involved specifically the procedures facelift, rhinoplasty, breast augmentation, augmentation mastopexy, mastopexy, and capsulectomy. To evaluate the influence of the fellowship on their subsequent professional orientation, participants were asked about scope of daily practice (Q23), number of (Q16–17, Q20) and confidence with aesthetic surgeries (Q22), as well as interest in and experience with research after graduating from the fellowship program (Q25, Q27). Finally, graduates were asked if they believe the fellowship had accelerated their career (Q29) and if they would recommend the fellowship in general (Q30).

### Statistics

For statistical analysis, we used the statistical package for social sciences (SPSS Version 11.5, IBM SPSS, Chicago, IL). Results are presented as mean ± standard deviation (SD) or median for continuous variables and as absolute numbers or percentage for categorical variables. We applied a student´s *t* test for unpaired samples to test for significant differences between means of the groups. Categorical variables were compared with a Chi-square test. *p* values were considered statistically significant at *p* < 0.05.

## Results

Thirty-four graduates participated in the survey (52%). Thirty were male and four female (12%). Mean age was 38.5 years (range 30–48years). Participants came from Australia (*n *= 1), Austria (*n *= 1), Cyprus (*n *= 1), Iceland (*n *= 1), Litany (*n *= 1), Montenegro (*n *= 1), Romania (*n *= 1), San Marino (*n *= 1), Turkey (*n *= 1), India (*n *= 1), Brazil (*n *= 2), Sweden (*n *= 2), Germany (*n *= 4), Greece (*n *= 5), United Kingdom (*n *= 5), and Italy (*n *= 6).

Twenty-four graduates (71%) had been already specialists in plastic surgery before commencing the fellowship program, while 29% were in their 4th or 5th year of plastic surgery residency.

Time from end of fellowship to conduction of survey was 43 months on average (range 3 to 120 months). Scope of daily clinical practice before the fellowship (multiple selections possible) was reconstruction in 26 cases (76%), aesthetics in 19 cases (56%), hand surgery in 18 cases (53%), burns in 8 cases (24%) and others in four cases (12%; microsurgery, oncologic surgery, trauma surgery). The fellowship was financed by (multiple selections possible) Akademikliniken in 22 cases (65%), savings in 19 cases (56%), home clinic/practice in 5 cases (15%), and debt in three cases (9%). Main motivation to apply for the fellowship was interest in aesthetics in 29 cases (85%) and additional research in 5 cases (15%). No Fellow had a sole interest in research only. Mean length of fellowship was 7 months (range 3–24months).

During fellowship the most interesting field for participants was breast in 26 cases (76%), face (without nose) in 4 cases (12%), nose in two cases (6%), body contouring in one case (3%), and botox/fillers in one case (3%). The most common surgery graduates participated in during their fellowship was breast augmentation (59% of graduates participated in more than 50 cases), followed by reduction mammaplasty/mastopexy (44% of graduates participated in 20–49 cases), augmentation-mastopexy (35% of graduates participated in 20–49 cases), facelift (53% of graduates participated in 5–19 cases), capsulectomy (50% of graduates participated in 5–19 cases), rhinoplasty (50% of graduates participated in 5–19 cases), blepharoplasty (47% of graduates participated in 5–19 cases) and brow lift (41% of graduates participated in 5–19 cases).

After the fellowship, twenty-four graduates (71%) changed their institution within one year, two (6%) within two years and two (6%) within more than two years after completion of the fellowship program. Six graduates did not change their institution after the fellowship (18%). At the time point of survey conduction, all but two participants were board certified (94%). Twenty-two graduates (65%) worked in a private practice, nine in a mixed private practice/hospital (26%), four in a hospital as attending (12%) and three in a hospital as resident (9%). Sixty-four percent of participants implemented more than 50% of surgical techniques learned during fellowship into their current daily surgical practice.

Numbers of aesthetic procedures before fellowship involved less than 50 aesthetic surgeries per year in 30 participants (88%), 51 to 100 in three (9%) and 201 to 500 in one (3%) participant. After the fellowship, 15 graduates (44%, *p* < 0.05) undertook less than 50 aesthetic surgeries per year, six (18%) 51 to 100, seven (21%; *p* < 0.05) 101 to 200, five (15%; *p* < 0.05) 201 to 500 and one (3%) more than 500. 6 graduates (18%) reported that the amount of aesthetic surgeries in their current daily practice more than doubled, 3 (9%) that it doubled, 7 (21%) that it increased by < 50%, 9 (26%) that it increased by < 25%, 8 (23%) that the amount remained the same and only one graduate (3%) reported that the amount decreased.

Before the fellowship, average confidence ratings were 1.6, 3.2, 2.0, 3.0, 3.0, 2.4, 3.3, 3.4, 2.8, and 3.4 for facelift, blepharoplasty, rhinoplasty, breast augmentation, mastopexy, augmentation mastopexy, breast reduction, abdominoplasty, brachioplasty, and liposuction, respectively. After fellowship average confidence ratings increased to 3.2, 4.3, 3.1, 4.5, 4.2, 4.0, 4.4, 4.6, 4.0, and 4.4 for facelift (*p* < 0.05), blepharoplasty, rhinoplasty (*p* < 0.05), breast augmentation, mastopexy (*p* < 0.05), augmentation mastopexy, breast reduction, abdominoplasty, brachioplasty (*p* < 0.05), and liposuction, respectively.

Scope of daily clinical practice before the fellowship (multiple selections possible) was reconstruction in 26 cases (76%), aesthetics in 19 cases (56%), hand surgery in 18 cases (53%), burns in 8 cases (24%) and others in four cases (12%; microsurgery, oncologic surgery, trauma surgery). After the fellowship, the scope of clinical practice was reconstruction in 18 cases (53%), aesthetics in 28 cases (82%; *p* < 0.05), hand surgery in 5 cases (15%), burns in 0 cases (0%) and others in three cases (9%).

In terms of scientific work, 17 (50%) graduates reported that they had published < 5 papers before the fellowship, 9 (26%) had published between 5 and 10 papers, 7 (21%) had published 10–30 papers, one (3%) had published 30–50 papers and none had published above 50 papers. After the fellowship 11 (32%), 11 (32%), 9 (27%), 2 (6%) and 1 (3%) reported having published < 5, 5–10, 10–30, 30–50 and > 50 papers, respectively. Of all graduates, 14 (41%) reported that the interest in research increased, while the interest did not alter in 18 cases (53%). Only 2 (6%) graduates reported a decreased research interest after the fellowship.

In terms of fellowship evaluation, most graduates (33, 97%) stated that the fellowship fulfilled their expectations while only one (3%) reported otherwise. 29 (85%) former fellows thought that the fellowship accelerated their career while 5 (15%) answered with no to this question.

Overall, 33 of 34 graduated (97%) would recommend the fellowship to other colleagues.

## Discussion

Over the past years, the demand for cosmetic surgical and non-surgical procedures has risen by 27% and 38% [2]. Simultaneously, the plastic surgery training environment constantly progressed with a variety of different integrated programs and fellowship opportunities. Due to the increasing demand of patients for aesthetic procedures, a more in-depth education and emphasis on both, reconstructive and aesthetic surgery is warranted during the plastic surgery training [10].

In a recent study about the quality of aesthetic surgery training among German residents, Momeni et al. found that 69% of 112 participants reported, that no aesthetic training is provided during their residency [11]. Moreover, 88% stated that no dedicated cosmetic surgery rotation was available. This ties in with Hashmi et al., who conducted a survey of the current state of plastic surgery training [3]. This survey evidences that besides a separate rotation in microsurgery, aesthetic surgery was the least offered rotation during residency programs. In fact, when asked to specify the specialty they were least trained in, most residents most commonly reported aesthetic surgery as such. However, more than 50% of residents in a survey by Ngaage et al. reported that cosmetic surgery training was an important factor in deciding for a residency program [2]. This is contrasted by the planned career paths among plastic surgery residents. Senior residents in plastic and reconstructive surgery were asked about their future career plans in a study by Imahara et al. [12]. The overwhelming majority planned to go into a solo or group private practice with a focus on aesthetic surgery instead of staying in an academic hospital. During our survey among graduates of our aesthetic surgery fellowship program, we found similar results. While 77% of graduates changed their institution within two years, the most common occupation after the fellowship was in a private practice (65%) or a mixed private practice/hospital (26%).

The lack of adequate training in cosmetic procedures during residency and the aim to go into private practice arise the need for dedicated aesthetic surgery fellowships after residency in order to guarantee an adequate patient treatment. In a large-scale survey among plastic surgeons with 600 participants, a total of 290 (48.3%) reported to have completed a fellowship training program after residency [13]. Yet, only 46 study participants (7.7%) completed a dedicated aesthetic surgery fellowship at the timepoint of the survey. This low rate was, at least partly, contributed to the lack of dedicated aesthetic fellowship opportunities.

Three key factors define the problems in aesthetic surgery: (1) private patient population with the demand to be operated by an experienced plastic surgeon, (2) lack of a dedicated curriculum during training and (3) a shortage of aesthetic procedures at academic hospitals [3].

Addressing the first problem, devoted resident aesthetic surgery clinics are on the rise in the USA in which both, residents and patients, can benefit from the unique setting. However, these resident surgery clinics are not yet widespread found and not yet very popular in Europe. Aesthetic surgery fellowships, as provided by our institution, are a good tool to address the other key problems in aesthetic surgery education.

The confidence level in a subspeciality training is a direct product of experience in that field. Hashmi et al. found that after approximately 6.4 months of training, residents felt comfortable in their subspeciality, whereas graduates who only spent 3 months felt the least confident [3]. Our questionnaire revealed that graduates of the fellowship program at Akademikliniken Stockholm spent on average 7 months on the training in aesthetic surgery. Fellowship graduates were asked to rate their confidence level in major aesthetic procedures before their fellowship training. On a Likert scale from 1 (not confident) to 5 (confident), mean confidence levels were 1.6, 3.2, 2.0, 3.0, 3.0, 2.4, 3.3, 3.4, 2.8, and 3.4 for facelift, blepharoplasty, breast augmentation, mastopexy, augmentation/mastopexy, breast reduction, abdominoplasty, brachioplasty, and liposuction, respectively. This ties in with the results of Momeni et al. who found that none of the senior plastic surgery residents claimed to be very confident and only 34% felt confident in performing those procedures [11]. Especially for facelifts, residents felt a need for improved education. In this regard, our fellowship could increase the mean confidence rating of graduates in facelifts to 3.2, while increasing the confidence levels for blepharoplasty, breast augmentation, mastopexy, augmentation/mastopexy, breast reduction, abdominoplasty, brachioplasty, and liposuction to 4.3, 3.1, 4.5, 4.2, 4.0, 4.4, 4.6, 4.0, and 4.4, respectively. These increases showed to be statistically significant for facelifts, rhinoplasty, mastopexy and brachioplasty.

A study indicated that only half of graduating plastic surgeons feel comfortable implementing cosmetic surgery into their daily practice highlighting the importance of dedicated fellowship programs, like provided by our institution, to boost experiences and confidence with aesthetic surgery [14]. This is even more important in the light of the highly competitive field of aesthetic surgery, which is offered by a variety of different non-plastic surgery specialties. In this context, improving the aesthetic education of plastic surgery residents, means improving the overall standing of plastic surgeons as the main provider for aesthetic procedures. Accordingly, studies focusing on medical tourism for aesthetic procedures did reveal very high rates of infective complications and return to theater rates in patients with aesthetic breast surgeries [15]. In many European countries aesthetic procedures are frequently performed by non-plastic surgeons and in most countries there is no current law, demanding a real education in order to be allowed to perform aesthetic surgery procedures. In the era of Social Media, where it is easy to attract patients just by pretending to be an expert even without being one, a better regulation should be needed to help patients choose their surgeon.

The presented survey study has some limitations, which need to be addressed.

The study included 34 participants in total, which is a fairly limited number to draw definitive conclusions. However, while other survey studies yielded a respondent rate of 13–30%, participation in our questionnaire was rather high with 52% of all invited graduates. Furthermore, the questionnaire was designed to inquire about the graduates’ demographics, surgical skills before and after the fellowship as well as their experience during their fellowship. The questionnaire itself has been designed by the authors and did not go through a strict validation process.

Another limitation of this study is that analysis of survey results is, except for some parameters, descriptive and does not offer the possibility for statistical analysis. Yet, the presented results offer a beneficial insight in the perception of dedicated aesthetic surgery fellowships by plastic surgeons in training and emphasize the need for implementation of adequate training in the plastic surgery curriculum.

## Conclusion

The results of the present study show that the lack of cosmetic surgery training during residency can be overcome by a well-designed dedicated aesthetic surgery fellowship. Graduates of our fellowship program reported great improvements in confidence in performing aesthetic procedures and a benefit for their future career.
