# Correction: Aesthetic Surgery Fellowship at Akademikliniken Stockholm: Survey among Graduates

**DOI:** 10.1007/s00266-024-04467-3

**Published:** 2024-10-23

**Authors:** Paolo Montemurro, Yannick F. Diehm, Per Hedén, Sebastian Fischer, Johannes M. Wagner

**Affiliations:** 1 Akademikliniken Stockholm, Storängsvägen 10, 11541 Stockholm, Sweden; 2 Department of Surgery, Division of Plastic Surgery, Brigham and Women’s Hospital, Harvard Medical School, 75 Francis St, Boston, 02115 USA; 3 Department of Plastic and Reconstructive Surgery, BG Clinic Ludwigshafen, University of Heidelberg, Ludwig-Guttmann-Straße 13, 67071 Ludwigshafen, Germany; 4 Department of Plastic, Reconstructive and Hand Surgery, BG University Hospital Bergmannsheil Bochum, Bürkle-de-la-Camp Platz 1, 44789 Bochum, Germany

**Correction to: Aesthetic Plast Surg . 2024 Mar 27.** 10.1007/s00266-024-03939-w

The article “Aesthetic Surgery Fellowship at Akademikliniken Stockholm: Survey among Graduates”, written by Johannes Wagner et al, was originally published electronically on the publisher’s internet portal on 27 March 2024 without open access. With the author(s)’ decision to opt for Open Choice the copyright of the article changed on 10 October 2024 to © The Author(s) 2024 and the article is forthwith distributed under a Creative Commons Attribution CC BY: This licence permits use, duplication, adaptation, distribution and reproduction in any medium or format, as long as appropriate credit is given to the original author(s) and the source, a link is provided to the Creative Commons licence, and any changes made are indicated. Please read the full licence for further details at https://creativecommons.org/licenses/by/4.0/.

